# Comparison between Chondrogenic Markers of Differentiated Chondrocytes from Adipose Derived Stem Cells and Articular Chondrocytes *In Vitro*

**Published:** 2013-06

**Authors:** Mohmmad Mardani, Batool Hashemibeni, Malek Masoud Ansar, Sayeed Hamid Zarkesh Esfahani, Mohmmad Kazemi, Vahid Goharian, Nafiseh Esmaeili, Ebrahim Esfandiary

**Affiliations:** 1 Department of Anatomical Sciences and Molecular Biology, Medical School, Esfahan University of Medical Sciences, Esfahan, Islamic Republic of Iran; 2 Department of Immunology, Medical School, Esfahan University of Medical Sciences, Esfahan, Esfahan, Islamic Republic of Iran; 3 Amin Hospital, Medical School, Esfahan University of Medical Sciences, Esfahan, Esfahan, Islamic Republic of Iran; 4 Department of Immunology, Medical School, Esfahan University of Medical Sciences, Esfahan, Islamic Republic of Iran

**Keywords:** Alginate, Stem cell, Chondrocyte, TGFbeta

## Abstract

***Objective(s):*** Osteoarthritis is one of the most common diseases in middle-aged population in the world. Cartilage tissue engineering (TE) has been presented as an effort to introduce the best combination of cells, biomaterial scaffolds and stimulating growth factors to produce a cartilage tissue similar to the natural articular cartilage. In this study, the chondrogenic potential of adipose derived stem cells (ADSCs) was compared with natural articular chondrocytes cultured in alginate scaffold.

***Materials and Methods:*** Human ADSCs were obtained from subcutaneous adipose tissue and human articular chondrocytes from non-weight bearing areas of knee joints. Cells were seeded in 1.5% alginate and cultured in chondrogenic media for three weeks with and without TGFβ3. The genes expression of types II and X collagens was assessed by Real Time PCR and the amount of aggrecan (AGC) and type I collagen measured by ELISA and the content of glycosaminoglycan evaluated by GAG assay.

***Results:*** Our findings showed that type II collagen, GAG and AGC were expressed, in differentiated ADSCs. Meanwhile, they produced a lesser amount of types II and X collagens but more AGC, GAG and type I collagen in comparison with natural chondrocytes (NCs).

***Conclusion:*** Further attempt should be carried out to optimize achieving type II collagen in DCs, as much as, natural articular chondrocytes and decline of the production of type I collagen in order to provide efficient hyaline cartilage after chondrogenic induction, prior to the usage of harvested tissues in clinical trials.

## Introduction

Based on the current statistics, millions of people in the world suffer from articular diseases which are associated with joint destruction (1-3). Traditional treatments, despite some successes, have not yet been satisfactory (4, 5). Thus tissue engineering which is a combination of engineering principles and biological sciences has been considered to have an important role in the treatment of articular lesions (6). Tissue engineering is seeking for cellular biomaterial scaffolds and appropriate stimulating growth factors to produce alternative hyaline articular cartilage that could be transplanted in order to treat articular defects (7-10). 

The selection of suitable cells is often difficult, as cells should be autologous (not responsive immunologically), having high proliferative power and potentially differentiable to the desired target cells (11). Autologous chondrocytes are the first differentiated cells used for cartilage tissue engineering, then neonatal (12) and finally fetal (13) chondrocytes. Due to some limitations of using mentioned cells, adult undifferentiated stem cells with high differentiation ability to chondrocytes, have been considered as an alternative. Among different sources of stem cells, those of adipose derived stem cells (ADSCs) and bone marrow derived mesenchyme stem cells (BM-MSCs) have been used in cartilage tissue engineering (15-17). In recent years due to ease of accessibility to subcutaneous adipose tissue and availability of large quantity of cells compared with the bone marrow, ADSCs have received a great interest (18-20). So far, the characteristics of ADSCs have not been well defined and more investigation is necessary (20, 21).

Another important factor in TE is selection of proper biomaterial scaffold which should have biocompatibility, biodegradability and facilitator of tissue proliferation and proper biomechanical properties (23, 24). In TE both hydrogels and porous scaffolds are used. Alginate is a kind of natural hydrogel which is derived from seaweed. Many researchers have investigated this hydrogel as an injectable seeded chondrocytes scaffold for articular cartilage repairmen (25-27). Previous studies have indicated that differentiated chondrocytes from ADSCs in alginate were morphologically spherical and similar to natural chondrocytes. Moreover, differentiation trend requires other suitable growth factors as well as scaffolds such as TGFβ, FGF, IGF and BMP (1). Among these, TGFβs including TGFβ1, TGFβ2, TGFβ3 and BMPs have the most potential for differentiation induction to chondrocytes in MSCs. It is reported that TGFβ2, TGFβ3 are more effective in induction of human MSCs to chondrocytes than TGFβ1 (30). In the past two decades except few cases no suitable cartilage tissue has been designed in vitro for clinical applications (14, 31). Therefore, it seems that TE is still at its infancy and in order to address many clinical issues, a long way should be followed. It is hoped that, by clarifying ambiguities such as, precise mechanisms of differentiation of stem cells to the chondrocytes, the pattern of interaction with various scaffolds, further determination of differences between articular chondrocytes. Moreover, the differentiated chondrocytes from stem cells, a functional long life engineered cartilage tissue similar to articular cartilage would be presented to the medical fields (1, 29).

Many scientists have compared chondrogenesis between articular chondrocytes and chondrocytes derived from stem cells. Gleghorn *et al* (2005) and Mauck *et al* (2006), announced that mechanical properties and matrix extent produced by BM-MSCs derived chondrocytes in alginate is lower than the articular ones (32, 33). Jakobsen *et al *(2009) stated that type II collagen in BM-MSCs and ADSCs was equal to the natural chondrocytes in hyaluronic acid scaffold (34). In addition, Tigli *et al *(2009) revealed that the expression of chondrogenic markers, in embryonic stem cells, BM-MSCs, ADSCs and MSCs derived from embryonic stem cells improved in silk and chitosan than articular chondrocytes (35). Mahmoudifar *et al *(2010) indicated the higher chondrogenic potential in fetal chondrocytes compared with ADSCs in polyglycolic acid (PGA) (13). Despite such investigation on differentiation of ADSCs to chondrocytes and articular chondrocytes in alginate, no reported comparison between these cells has been made. Therefore, we aimed to assess and compare types II, X, I collagens, aggrecan and glycosaminoglycan (GAG) of differentiated chondrocytes from ADSCs, with natural articular chondrocytes seeded in alginate with and without TGFβ3 on days 14 and 21. 

## Materials and Methods

***Isolation and proliferation of ADSCs***

Written consent was obtained from all patients in an operation room. Subcutaneous adipose tissue (∼10 g) was obtained from 4 patients (30-50 years age), under sterile conditions and transferred to the lab. Afterward, it was digested with 0.075% collagenase type I (Sigma) at 37°C for 30 min. The enzyme was inactivated by DMEM LG (Sigma) containing 10% FBS (Invitrogen). Subsequently, the resultant solution was centrifuged (1200 rpm, 15 min) and cell pellet was cultured in 25 cm^2 ^flasks with DMEM LG, 10% FBS, 1% penicillin & streptomycin (Gibco) and incubated with 5% CO_2_, 37°C. The medium was changed every 4 days. , Cells were detached with 0.05% trypsin/0.53 mM EDTA (Sigma) and passaged, when they reached 80% confluence. P3-p5 cells were seeded in an alginate scaffold.

***Isolation& proliferation of a*** ***rticular chondrocytes***

After taking a written consent, a bit of articular cartilage was obtained by the knee arthroscopy from 4 patients (20-30 years age) whose joints did not show any signs of a degenerative arthritis. The specimens were taken from non-weight bearing areas and transferred within PBS (Sigma) to the lab. The specimens were then divided into 1×1 mm slices and digested with collagenase type II (Sigma) 350 u/ml in 37° for 4 hr. After inactivation with DMEM F12 (Gibco) with 10% FBS, the solution was centrifuged and cell pellets were cultured in 25 cm^2^ flask with DMEM F12, 10% FBS, 1% penicillin and streptomycin. When cells reached 80% confluence, they were detached with trypsin/EDTA and passaged. P2-P4 cells were used for seeding in an alginate scaffold.

***Encapsulation and culture procedure in alginate***

Initially, ADSCs (P3-P5) and articular chondrocytes (p2-p4) were separately resuspended in 1.5% alginate (Sigma) at 5×10^6^ cells/ml. The alginate/cell suspension was expressed through a 23-gauge needle into a 102 mM CaCl_2 _solution (Merck). The alginate beads after 15 min were washed twice in 0.9% saline solution and once in DMEM-HG (Gibco), and finally 2 ml chondrogenic media was added to each well of 12-well. Chondrogenic culture media contained: DMEM-HG (High Glucose)(Gibco), penicillin & streptomycin 1% (Gibco), dexamethasone 10^-7^M (Sigma), ascorbat-2-phosphate 50 µg/ml (Sigma), bovine serum albumin 1% (Sigma), linoleic acid 5µg/ml (Sigma), insulin-transferrin-selenium (ITS) 1% (Sigma),with and without adding transforming growth factor- β_3 _(TGFβ_3) _10 ng /ml (Sigma)

The plates were incubated with 5% CO_2 _at 37° and replaced every 4 days. Supernatant mediums on days 14 and 21 were frozen at -20° for ELISA.

***ELISA***

AGC and type I collagen produced in supernatant media with and without TGFβ_3_ on days 14 and 21 was measured by ELISA. For AGC the kit was from Invitrogen (Cat No KAP1461) and type I collagen was from MD bioproducts (Cat No M036007) according to their manufacturers’ protocols. Finally samples were read in ELISA reader (Hyperion, Microreader 4 plus) with 450 nm wave length.

***Biochemical assays***

***DNA quantification***

In order to determinate DNA and GAG , first, alginate beads on days 14 and 21 digested for 18 h at 60° in papain solution 125µg/ml(Sigma) containing cistein10 mM (Sigma) in PBE buffer (Na_2_Hpo_4_ 100 mM, EDTA 10 mM, pH=6.5). For each 12 beads, one ml enzyme was used. The resultant solutions were used for determination of DNA and GAG.

The content of DNA (ng/ml) was measured using DNA Quantification Kit, Fluorescence Assay (Sigma, Cat. No. DNAQF), according to its manufacturer’s protocol. Briefly, resultant solution was read by dyed Hoechst 33258 and absorbance of each sample was read by spectrofluorometer (Perkin Elmer LS-3) at 360 nm in excitation and 460 nm wave length in emission. Afterward, the DNA content was calculated according to the thymic calf standard curve.

***GAG Assay***

The amount of GAG (µg/ml) was quantified using 1, 9-dimethylmethylene blue (DMMB) dye. Briefly, 100μl of resultant solution was mixed with 2400 μl of DMMB solution in a cuvette and then its absorbance in 525 nm wave length was read by spectrophotometer (Spectonic 70, Baucsh&lomb). The GAG content was calculated using standard curve chondroitin sulfate of bovine trachea.

Finally, the ratio of GAG/DNA in each sample was normalized. All experiments were performed twice.

***Real-time PCR***

At first, alginate beads on days 14 and 21 were washed with PBS and for digestion, they were placed in 1.5% 55 mM citrate sodium (Sharlau) and 0.15 mM NaCl (Merck). The resultant solution centrifuged for 10 min at 1200 rpm and the derived cell pellet was used for the extraction of RNA with RNeasy mini kit (Qiagen,Cat. No. 74101) with a little modification (36). For lysing cells, firstly a solution mixed of 990 µl Trizol (Invitrogen) and 10 µl of 2-mercaptoethanol (Sigma), was kept in room temperature (RT) for 5 min. Next, 200 µl chloroform was added and shacked vigorously for 15 second kept in RT for 2-3 min and then centrifuged for 15 min at 4° at 12000 g.

The supernatant aqueous phase was transported into a 1.5 ml microtube and the same volume of ethanol 70% was added and then mixed. The resultant solution transferred to columns in kit and the rest of instruction was carried out according to kit protocol in which to kit protocol in which RNase free DNase set (Qiagen) applied for elimination of possible DNA contamination. The extent of derived RNA was measured by spectrophotometer (Biophotometer, Eppendorf) at 260/280 nm wave length. Reverse transcription for cDNA, 100 ng RNA used by recruitment of RevertAid^TM^ First Strand cDNA Synthesis Kit (Fermentas,Cat. No. #K 1621) according to manufacturer’s protocol.

Relative quantification of the expression types II and X collagens was measured, using Maxima SYBR® Green/RoxqPCR master Mix 2X (Fermentas), with GAPDH primer as an internal control. The calculation was performed via comparative Ct (ΔΔ Ct). The reactions conducted in 20 µl with, 10 µl SYBR® Green, 7.5 µl H_2_o, 0.25 µM forward and reverse primers and 1.5 µl cDNA as following planned by StepOne Plus Real Time PCR system (Applied Biosystem): primary denaturation in 95° for 10 min, denaturation in 95° for 15 sec, Annealing and Extension in 60° for 1 min –the whole process was done 40 cycles- and finally melt curve (increment 0.3 °C, 60°C→95°C) was depicted. All experiments were performed in triplicates for each specimen. The applied primers for Real-Time PCR were designed by AlleleID 7/6 software which indicated in Table 1.

***Statistical tests***

Kolmogorov-Simonov test was used for assessing the normal distribution of variables. ANOVA (one-way-analysis of variance) with LSD *post hoc* test used for comparison of ELISA, Real-Time PCR and GAG assay results in different groups.

## Results

***Gene expression of type II and X collagens***

The results of Real-Time PCR indicated that type II collagen gene expression in articular chondrocytes (NCs) is significantly higher (*P*<0.001) than differentiated chondrocytes (DCs) after two weeks. 

**Table 1:** Primers used in Real Time PCR

Gene	Primer sequences	Size (Base pair)
collagen II-F	CTGGTGATGATGGTGAAG	130
collagen II -R	CCTGGATAACCTCTGTGA	
collagen x –F	AGAATCCATCTGAGAATATGC	187
collagen x - R	CCTCTTACTGCTATACCTTTAC	
GAPDH-F	AAGCTCATTTCCTGGTATG	125
GAPDH-R	CTTCCTCTTGTGCTCTTG	

**Table 2:** Results of chondrogenic markers in differentiated chondrocytes (DCs) and articular chondrocytes (NCs) on days 14 and 21

Groups	Collagen II (RQ)^1^	Collagen X (RQ)	Collagen I μg/ml	Aggrecan ng/ml	GAG/DNA μg/ng
+DCs(14)	18.34	126.26*	0.316	28.65333*	0.35*
-DCs(14)	1.275	5.415	0.575	19.06333	0.095
+NCs(14)	195.16**	0.0013	0.27	14.5	0.3
-NCs(14)	457.4505*	0.0003	0.2102	15.03333	0.24
+DCs(21)	34.19	0.1759	1.0612*	13.46667	0.55**
-DCs(21)	4.03	2.73	0.5167	19**	0.19
+NCs(21)	3.675	750.8**	0.6697**	16.26667	0.24
-NCs(21)	13.465	87.35	0.2425	14.1	0.21
					

Collagen II: *P<0.001 vs. other groups on day 14. **P<0.001 vs. DCs groups on day 14. Collagen X: *P<0.05 vs. –DCs and NCs groups on day 14. **P<0.001 vs. other groups on days 14&21. Collagen I: *P<0.05 vs. all other groups on day14&21. **P<0.05 vs. –NCs on day 21 and NCs groups on day 14. Aggrecan: *P<0.001 vs. all other groups on days 14 &21. **P<0.001 vs. all other groups on day 14&21. GAG: *P<0.001 vs. -DCs and NCs groups on day 14. **P<0.001 vs. all other groups on days 14 &21

1= Relative Quantity

**Figure 1:**
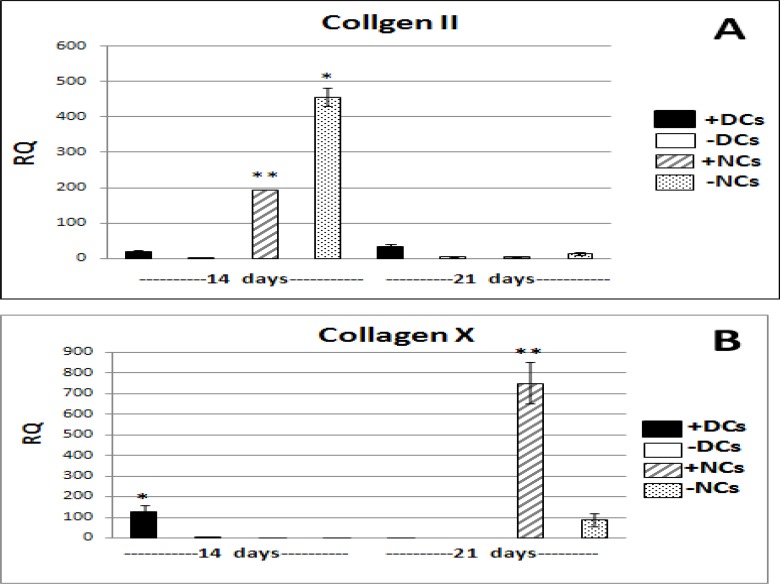
Results of Real Time PCR for collagens type II and type X in differentiated chondrocytes (DCs) and natural articular chondrocytes (NCs) with and without TGFβ3 on days 14 & 21. Values are the means +SE of triplicate experiments.

TGFβ3 resulted in a significant (*P*<0.001) decrease of type II collagen expression in NCs, but its expression reduced strongly in both groups of NCs at the end of third week. Also, TGFβ3 caused increase in type II collagen gene expression in DCs in third week than in second week. Nevertheless, DCs expressed the lowest amount of type II collagen in second and third weeks. (Figure 1, A) (Table 2).

The highest amount of type X collagen gene expression, a hypertrophic factor, was seen in articular chondrocytes (NCs) in third week which was significantly (*P*<0.05) more than differentiated chondrocytes (DCs), while it was not expressed in these cells in the second week. Adding TGFβ3 led to significant increase of gene expression in third week (*P*<0.001) compared to NCs without TGFβ3. Also, DCs in the presence of this growth factor significantly (*P*<0.05) expressed type X collagen higher than other group without it at the end of second week. Generally, with the course of time and in third week DCs no longer expressed this gene (Figure 1, B) (Table 2).

(A)*P<0.001 vs. other groups on day 14. **P<0.001 vs. DCs groups on day 14. **(B)** *P<0.05 vs. –DCs and NCs groups on day 14. **P<0.001 vs. other groups on days 14&21.

+DCs, Differentiated chondrocytes with TGFβ3; -DCs, Differentiated chondrocytes without TGFβ3;+NCs, natural articular chondrocytes with TGFβ3; -NCs, natural articular chondrocytes without TGFβ3

**Figure 2:**
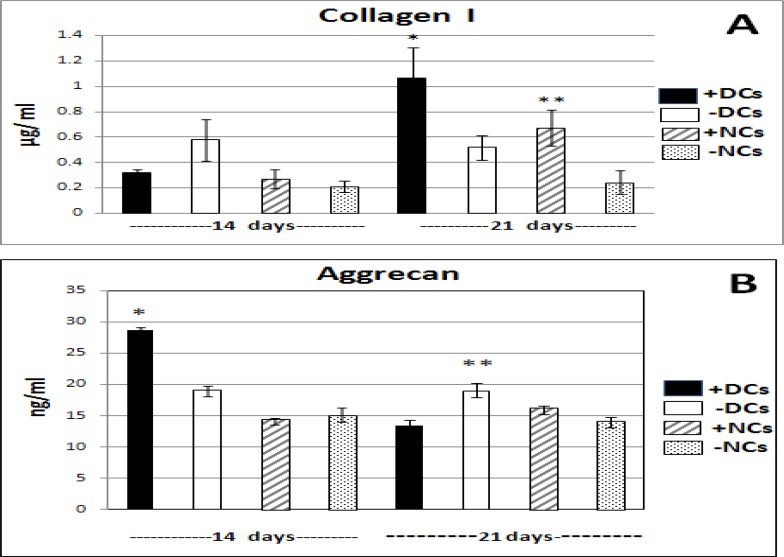
The results of ELISA analysis for collagen type I and aggrecan in supernatant media of DCs and NCs with and without TGFβ3 on days 14 & 21.Values are the means +SE of triplicate experiments.

**Figure 3:**
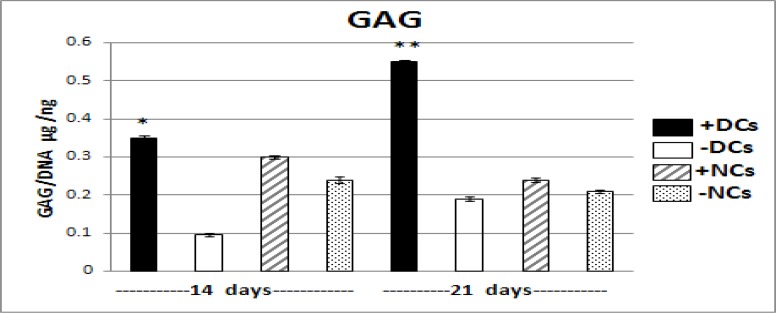
The GAG content in DCs and NCs with and without TGFβ3 on days 14 & 21.

Values are the means +SE of duplicate experiments.*P<0.001 vs. -DCs and NCs groups on day 14. **P<0.001 vs. all other groups on days 14 &21.+DCs, Differentiated chondrocytes with TGFβ3; -DCs, Differentiated chondrocytes without TGFβ3; +NCs, natural articular chondrocytes with TGFβ3; -NCs, natural articular chondrocytes without TGFβ3.

***Production of Aggrecan, Type I collagen and GAG***

The results of ELISA indicated that articular chondrocytes (NCs) produced aggrecan (AGC) constantly during second and third weeks and TGFβ3 had no effect on it. In general, differentiated chondrocytes (DCs) generated more AGC than NCs which was significantly different (*P*<0.001) in second week. In the presence of TGFβ3, DCs caused more significant production in second week but decreased in third week compared to other group without TGFβ3(*P*<0.001). (Figure 2, B) (Table 2).

In general, type I collagen production increased in all groups in third week in comparison to second week. However, articular chondrocytes (NCs) produced less type I collagen in two time points which showed significant difference (*P*<0.05) only in third week. Adding TGFβ3 to DCs and NCs yielded more type I collagen in third week indicating significant difference (*P*<0.05) compared to NCs and DCs without TGFβ3. (Figure 2, A) (Table 2).

The results of GAG assay indicated that in third week articular chondrocytes (NCs) produced glycosaminoglycan (GAG) less than second week but the trend of its production in differentiated chondrocytes (DCs) was significantly increased in third week compared to second week (*P*<0.001). In addition, TGFβ3 resulted in significant increase in GAG production than other groups without it in NCs and DCs in two time points in that the highest amount was related to DCs (*P*<0.001). Generally, DCs generated more GAG than NCs in second and third weeks (Figure 3) (Table 2). 

## Discussion

One of the aims of TE is to find a suitable alternative cell source to synthesize an extra cellular matrix similar to natural cartilage (35). In recent years, several researchers have studied chondrogenesis in ADSCs and have compared them with other cells in different scaffolds and various growth factors (17-20, 32 -35) but there is no detailed comparison between differentiated cells from ADSCs and natural articular chondrocytes in alginate. In this study, we compared differentiated chondrocytes from ADSCs and articular chondrocytes separately in alginate with and without TGFβ3 for 21 days. 

According to our findings, the gene expression of type II collagen, GAG production and their progressive increase during 21 days. In addition, the existence of aggrecan (AGC) in supernatant media as essential cartilage markers in differentiated chondrocytes (DCs), confirms that ADSCs have been differentiated to chondrocytes. Comparing results of these markers between DCs and articular chondrocytes (NCs) after two weeks, indicated that gene expression of type II collagen was significantly lower than NCs, which was in consistent with previous reports about BM-MSCs in agarose and ADSCs in polyglycolic acid (PGA)(33, 13). However, it was reported that in hyaluronic acid scaffold type II collagen expressed equally by ADSCs or chondrocytes and BM-MSCs expressed more than chondrocytes and the effect of BMP2 as well as TGFβ1 may have a role in this phenomenon. Unlike previous studies, our results indicated that DCs produced more AGC and GAG than NCs (33, 32).Tigli *et al *(2009) indicated that embryonic stem cells (ESCs), ESCs derived MSCs, ADSCs and BM-MSCs in silk and chitosan, expressed chondrogenic markers, such as type II collagen, Sox9 and AGC more than NCs (35) which is consistent with our findings about GAG and AGC but in contrast to type II collagen. This discrepancy may be due to the application of two growth factors, BMP2 and TGFβ1 at the same time and interaction between these factors and two other different scaffolds. Increasing level of GAG production in DCs and superiority to NCs and also gene expression of type II collagen and generation of AGC in third week indicates that DCs in alginate during three weeks will maintain its phenotype. Decrease of some chondrogenic markers in third compared to second week such as AGC in DCs or type II collagen and GAG production in NCs may be due to the fact that the highest amount of extracellular matrix was released on day14 and therefore the matrix molecules themselves caused reduction of these markers as a negative feedback and modification of chondrocyte metabolism (37, 38). On the other hand, gene expression of type X collagen–a hypertrophic factor in embryonic stage- (39) was considered as a big challenge during differentiation of BM-MSCs and ADSCs to chondrocytes (30, 40, 41) and chondrocytes redifferentiation in various scaffolds and pellet culture (35, 42, 43). In this study, type X collagen in DCs was expressed significantly more than NCs after two weeks, but reduced considerably and distinguished after three weeks. Its concomitant with type II collagen gene expression and GAG production increase and continuation of AGC production at this point are positive signals for differentiation.

Of course the gene expression of type X collagen is not necessarily accompanied with collagen X protein production and hypertrophy (44) which may be the sign of rapid maturation of these cells (42). Some studies have reported that, healthy adult chondrocytes in tissue engineering have not-or very little- expressed this gene (45-47). Other studies stated that, articular chondrocytes expressed less type X collagen, compared to ESCs-derived MSCs in silk and chitosan (35), ADSCs in hyaluronic acid (34) and pellet culture (48), which was in contrast with our present findings in the third week. However, in line with our results, it was reported gene expression of type X collagen by BM-MSCs in silk and chitosan was lower than articular chondrocytes (35). 

Types I and X collagens are considered as negative markers in chondrogenesis. It has been noted that MSCs especially ADSCs and BM-MSCs produce a lot of collagen type I (20, 49, 50). For TE, the expression of this gene is not appropriate and after *in vivo* implantation produces fibrocartilage instead of hyaline (13). Principally, scaffolds that maintain spherical shape of cells and prevent their contact to each other -like agarose and alginate- enhance chondrogenesis and inhibit production of collagen type I (22). Progressive increase of type I collagen production in all groups of our study during three weeks means that other factors may be involved other than scaffold and TGFβ3 *in vitro*. This gradual increase of type I collagen is in line with the report of Jakobsen *et al* (34) but in contrast with Yang *et al* (51). In consistent with our findings, it was reported that type I collagen in MSCs in the pellet culture, silk and chitosan scaffolds was produced more than NCs (35, 48). Conversely, it was reported that type I collagen in ADSCs and BM-MSCs in hyaluronic acid was equal to NCs (34).

The detection of AGC and type I collagen proteins in supernatant media by ELISA is due to lack of adequate structural consistency around the cells and washing away into the supernatant media. In a similar study, Jacobsen *et al* noted that despite high gene expression of type II collagen by BM-MSCs encapsulated in hyaluronic, immunohistochemistry technique did not show a large quantity of type II collagen in resultant tissue, while it was detectable in supernatant media by ELISA (34).

Transforming growth factor type beta (TGFβ) is the frequently- used growth factor for chondrogenesis *in vitro* which has a major role in initiation of chondrogenesis. A lot of studies indicated its provoking role in proliferation and generation of cartilage matrix in progenitor cells (52- 54). The review of literature denoted that TGFβ has different effects in chondrogenesis. Other studies have mentioned that type II collagen and GAG production were stimulated by TGFβ (55) and still some other groups have reported that it inhibits collagen and GAG production chondrocytes (56). TGFβ1 has a contribution in early stages (57) and TGFβ3 in chondrocyte maturation (58). It was reported that TGFβ3 increases gene expression of type II collagen in MSCs encapsulated in alginate (59), but Estes *et al *reported that TGFβ1 and TGFβ3 has no difference in proliferation, gene expression and cartilage biosynthetic activity in ADSCs in alginate (20). Moreover, the improvement of synthesis of chondrogenic markers such as AGC, link proteins, fibromodulin and decorin in MSCs and pellet culture in the presence of TGFβ3 has been clarified (16, 30). We found TGFβ3 improves chondrogenic markers such as AGC, GAG and type II collagen in DCs, which is alignment with some other previous reports (33, 55). However; it inhibits or has limited benefits in NCs which is in line with findings of Skantze *et al* (56) and in contrast with report of Mauck *et al *(33). Moreover, this growth factor has no effect in reducing of negative chondrogenic markers, types X and I collagens which is more prominent in third week. It was believed, TGFβ as a chondrogenic factor *in vitro*, causes retention of differentiated chondrocytes from MSCs in prehypertrophic state (60).Williams *et al* reported that TGFβ1 in MSCs, improved chondrogenic markers and inhibited collagen I (61), but Mahmoudifar *et al *stated that it had limited effect in cartilage synthesis (13). 

## Conclusion

ADSCs are multipotential cells easily obtainable from subcutaneous adipose tissue and so can be used as an alternative cell source for cartilage TE. According to our findings, despite low gene expression of type II collagen as a main chondrogenic marker, the trend gradually increased progressively from day 14 to 21. One reason is that more time may be required for ADSCs maturation after differentiation to chondrocytes compared to the natural articular chondrocytes (33).Type X collagen which is considered as a hypertrophic factor decreased during 21 days and possessed appropriate condition in DCs. However, type I collagen synthesis had a progressive trend that may lead to fibrocartilage instead of hyaline cartilage which is not suitable. It seems that more studies should be carried out to find new ways to produce type II collagen as much as possible and to inhibit type I collagen during chondrogenic differentiation of MSCs in various scaffolds in order to have efficient articular cartilage *in- vitro* to be used in clinics.
